# Energy-based constitutive modelling of local material properties of canine aortas

**DOI:** 10.1098/rsos.160365

**Published:** 2016-09-21

**Authors:** Kaveh Laksari, Danial Shahmirzadi, Camilo J. Acosta, Elisa Konofagou

**Affiliations:** 1 Department of Bioengineering, Stanford University, Stanford, CA, USA; 2 Department of Mechanical Engineering, Stevens Institute of Technology, Hoboken, NJ, USA; 3 Ultrasound and Elasticity Imaging Lab (UEIL), Department of Biomedical Engineering, Columbia University, New York, NY, USA; 4 Department of Radiology, Columbia University, New York, NY, USA

**Keywords:** aortic tissue, nonlinear hyperelastic constitutive modelling, histology

## Abstract

This study aims at determining the *in vitro* anisotropic mechanical behaviour of canine aortic tissue. We specifically focused on spatial variations of these properties along the axis of the vessel. We performed uniaxial stretch tests on canine aortic samples in both circumferential and longitudinal directions, as well as histological examinations to derive the tissue's fibre orientations. We subsequently characterized a constitutive model that incorporates both phenomenological and structural elements to account for macroscopic and microstructural behaviour of the tissue. We showed the two fibre families were oriented at similar angles with respect to the aorta's axis. We also found significant changes in mechanical behaviour of the tissue as a function of axial position from proximal to distal direction: the fibres become more aligned with the aortic axis from 46° to 30°. Also, the linear shear modulus of media decreased as we moved distally along the aortic axis from 139 to 64 kPa. These changes derived from the parameters in the nonlinear constitutive model agreed well with the changes in tissue structure. In addition, we showed that isotropic contribution, carried by elastic lamellae, to the total stress induced in the tissue decreases at higher stretch ratios, whereas anisotropic stress, carried by collagen fibres, increases. The constitutive models can be readily used to design computational models of tissue deformation during physiological loading cycles. The findings of this study extend the understanding of local mechanical properties that could lead to region-specific diagnostics and treatment of arterial diseases.

## 1. Introduction

Cardiovascular diseases are the leading cause of death and disability in the industrialized world with immense societal and economic burdens [1]. To alleviate the impact of such diseases, research has been performed in the regulation of cardiovascular systems and in particular the biomechanical response of arterial tissues to physiological and supra-physiological conditions [2]. Balloon-angioplasty and stenting are common procedures in treating atherosclerotic tissue [3,4]. As such, understanding and predicting the outcome of such procedures have achieved utmost importance in the medical field. Owing to the complexity of such procedures, computational models, especially finite-element (FE) modelling, have become an indispensable tool in design and optimization for diagnostic and therapeutic techniques.

On the one hand, FE models can accurately replicate geometrical variation of the arteries, a feat that has been performed precisely using modern imaging techniques. On the other hand, constitutive material models play a significant role in rendering the FE models able to accurately predict tissue behaviour. Establishing accurate, physiologically relevant models that are capable of describing the mechanical behaviour of aortic tissue is crucial in improving the design of medical devices. In addition, a direct relationship has been discovered between arterial stiffness and cardiovascular disease, which echoes the importance of mechanical properties of aortic tissue, both of healthy and pathological tissue, in terms of clinical diagnostics [5,6]. In particular, characterizing the complex mechanical behaviour of aortic tissue and incorporating the anisotropy, nonlinearity and phenomenological features are critical in establishing more accurate FE models.

The aorta consists of three layers: tunica intima, which is the innermost layer lining the lumen and is made of a single layer of endothelial cells; tunica media, which is the middle layer made of elastin lamellae, collagen and smooth muscle cells (SMC); and tunica adventitia, which is the outermost layer made of mostly circumferentially running collagen fibres, thin elastic fibres and fibroblasts [7]. The intima is very thin and does not contribute significantly to the mechanical behaviour of the aortic wall [8]. The media provides the main contribution to the elastic expansion and recoil of aorta (also known as the toe-region in the stress–strain curve), as well as blood pressure maintenance during systole and diastole. The adventitia mainly prevents excessive dilatation of the aortic wall and is less involved in lower deformation regimes due to collagen undulation [9]. The mechanical properties of the aorta depend heavily on the distribution and amount of elastin and collagen, with the compliant elastin being more prominent at lower pressures and deformations, and the stiff collagen taking more part in larger deformations [10,11].

The aortic tissue has been shown to exhibit a number of distinct mechanical behaviours including finite deformations and anisotropy even under physiological loads [12]. One of the most common methods to describe the complex material behaviour of soft tissues is through using energy-based constitutive models. Hyperelastic constitutive models have been used previously to describe the mechanical behaviour of arteries from physiological loading conditions to failure. These models include but are not limited to explicit stress–strain curve fits [13,14], isotropic Mooney–Rivlin models [3,15], and Fung's exponential orthotropic model, which is the most widely used constitutive model of arteries reported in the literature [16–18]. However, the above models do not incorporate the fibre-oriented structural elements of vascular tissues into the effect. More recently, an anisotropic model proposed by Holzapfel *et al.* [8,19,20] has been gaining more attention since it takes into account both phenomenological and structural measurements of the tissue. This family of constitutive models have been successfully implemented in commercial FE software, e.g. ABAQUS (Dassault Systemes) and LSDYNA (Livermore Software Technology Corporation), due to their continuum mechanical basis [21,22].

Changes in structural, geometrical and mechanical properties of aortas along the aorta's long axis were reported previously; however, such variations have not been fully quantified [14,23–27]. Accurate characterization of the local mechanical properties of the aortic tissue at different distal regions along the aorta is critical in designing diagnostic techniques such as those in endovascular aortic repair. In this study, we investigate the *in vitro* mechanical behaviour of canine aortic tissue under physiological and supra-physiological loading. Our model is characterized based on uniaxial stress–strain curves while incorporating fibre orientations obtained from histological images. Furthermore, we seek to characterize the anisotropic, local variations of mechanical properties of the tissue along its longitudinal axis.

## 2. Material and methods

### 2.1. Experimental set-up

#### 2.1.1. Sample preparation

**Figure 1.**
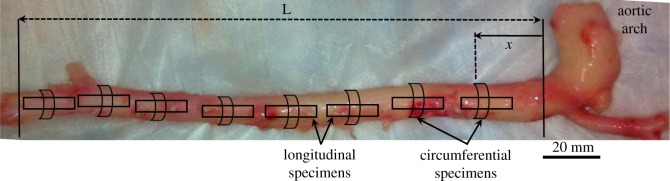
Representative image of a canine aorta indicating the normalized coordinate system: *x* and *L* are the axial location of each point under study and the total length of the excised aortic trunk, respectively. The schematics of excised circumferential and longitudinal specimens from multiple locations that were used for consequent mechanical testing are also shown in the figure.

Post-arch thoracic to infrarenal abdominal segments of the descending aortas were harvested from eight (*n* = 8) male mongrel dogs (weight 20–25 kg) immediately after euthanasia. Adipose and connective tissues were removed, and approximately *L* = 177.5 ± 23.5 mm long samples were excised. The aortic segments were kept in 4°C phosphate-buffered saline prior to cyclic loading experiments. A normalized coordinate system was established on each aorta in order to standardize the longitudinal locations for comparison among multiple specimens. In the normalized coordinate system, the distance from the proximal end (*x*) was divided by the total length (*L*) of the aortic segment (figure 1). In this definition, $\bar{x}=0$ indicates just below the aortic arch and $\bar{x}=1$ indicates the seventh intercostal artery.

#### 2.1.2. Uniaxial cyclic loading

Longitudinal and circumferential tissue specimens were extracted from each aorta sample at 0.1 normalized coordinate increments $\left(\bar{x}=0.1,0.2,\ldots ,0.9\right)$. Rectangular circumferential specimens (*n* = 48) of width *w* = 6.5 ± 1.2 mm, length *l* = 11.3 ± 3.2 mm and thickness *h* = 1.5 ± 0.3 mm, as well as longitudinal specimens (*n* = 13) of width *w* = 6.1 ± 1.4 mm, length *l* = 10.5 ± 3.0 mm and thickness *h* = 1.5 ± 0.3 mm were prepared. Local measurements of the thickness of the aortic wall were made non-invasively using image processing software ImageJ (NIH, Bethesda, MD, USA). For each thickness measurement, three separate readings were made and the average was reported. A total of 61 specimens were examined under uniaxial cyclic tensile testing using Instron 5848 micro-tester (Instron, Norwood, MA, USA). To precondition each excised tissue specimen, 20 cycles of tensile loading at strain and strain rate of ε=0.05 and $\dot{\varepsilon }=1\;s^{-1}$ were initially applied. The actual mechanical loading followed immediately as five cycles of tensile strain under ε=0.4 and $\dot{\varepsilon }=1\;s^{-1}$. The entire *in vitro* mechanical testing was completed within 24 h post-mortem so as to minimize tissue degradation.

#### 2.1.3. Histology

After completing the mechanical testing, the tissue specimens were used to extract 2 × 2 mm^2^ samples for histological examinations. Paraffin-embedded tissue processing was used to obtain different sections of the tissue from intimal, medial and adventitial layers across the wall thickness. Specimens were sectioned successively every 6 µm tangential to the circumferential-radial plane. A modified Masson's Trichrome Stain (with Fast Green) and Verhoeff's Hematoxylin was used to stain for elastin and collagen in black and green/blue, respectively, and additional components of proteoglycans, cytoplasm, keratin and muscle fibres in red/pink. Tissue specimens were also preheated in Bouin's solution at 56°C for 15 min in order to intensify the strains.

The aim here was to characterize the anisotropy and orientation distribution of each stained section and use this information in the constitutive equation given in the next section. In order to determine the fibre orientations, we used ImageJ (NIH) and an additional plug-in (OrientationJ) that calculates local orientation based on structure tensors [28,29]. OrientationJ determines the fibre directions in each area of interest by defining a weighted inner product and calculating the eigenvectors of the structure tensor, which is the partial spatial derivative of the image. We used at least 15 histology images at each location from each aortic specimen to determine the average fibre orientations.

#### 2.1.4. Constitutive modelling

**Figure 2.**
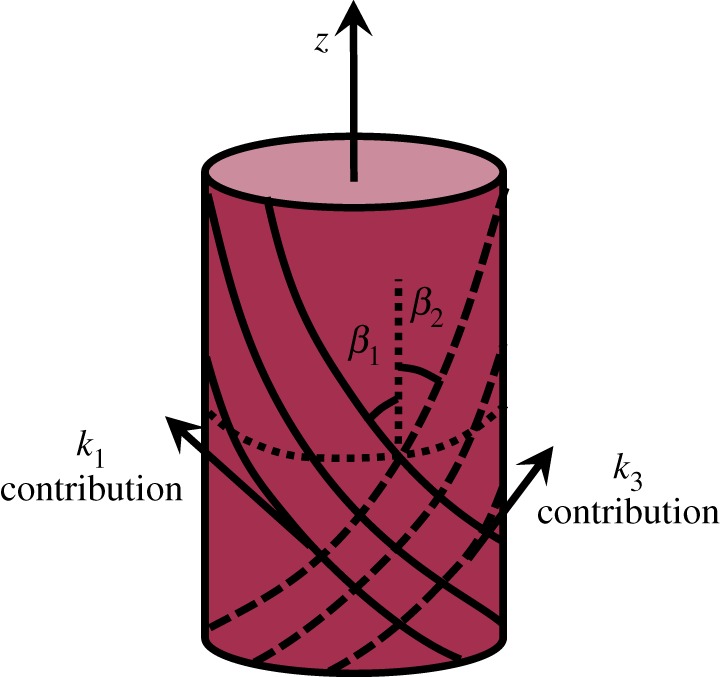
Schematic of the two families of fibres running helically along the aortic axis (*z*).

In this study, we assume the aortic tissue to be a composite material reinforced by two families of fibres in two preferred directions (figure 2). Therefore, we used the strain energy density function (SEDF) proposed by Holzapfel *et al.* [8,19]. The assumption here is that there are two contributions to the total stress induced in the tissue: an isotropic contribution and an anisotropic contribution, respectively, denoted by *iso* and *aniso* subscripts in the following equation:

2.1 $$\begin{array}{rl} W & =W_{\mathrm{iso}}+W_{\mathrm{aniso}}\\ & ={\left\{\mu (I_{1}-3)\right\}}_{\mathrm{iso}}+{\left\{\frac{k_{1}}{2k_{2}}(\exp(k_{2}{\left(I_{4}-1\right)}^{2}-1))+\frac{k_{3}}{2k_{4}}(\exp(k_{4}{\left(I_{6}-1\right)}^{2}-1))\right\}}_{\mathrm{aniso}}, \end{array}$$

where *W* is the SEDF with a dimension similar to stress, *I*_1_ is the first invariant of the right Cauchy–Green strain tensor (***C***), representing the isotropic contribution of the deformation. Right Cauchy–Green strain tensor is physically interpreted as the square of local change in length and is calculated by ***C*** = ***F***^T^***F***, where ***F*** denotes the deformation gradient tensor, whose diagonal elements are the stretch ratios (λ*_i_*) in the three cylindrical directions (*i* = *θ*, *z*, *r*) as depicted in figure 2. The mechanical contributions in the two preferred directions are represented by $I_{4}=\lambda_{z}^{2}\mathrm{co}s^{2}\beta_{1}+\lambda_{z}^{2}\sin^{2}\beta_{1}$ and $I_{6}=\lambda_{z}^{2}\mathrm{co}s^{2}\beta_{2}+\lambda_{\theta }^{2}\sin^{2}\beta_{2}$. Also, *µ* (the shear modulus) and *k*_1_ and *k*_3_ are stress-like parameters and *k*_2_ and *k*_4_ are dimensionless scalars. Constitutive equation (2.1) is equivalent to the more sophisticated model proposed in Holzapfel *et al.* [30] and used in García *et al.* [31] for the dimensionless dispersion factor *ρ* = 1, indicating full anisotropy in the tissue.

In the equation above, the preferred directions coincide with the directions of the fibre bundles, which are oriented around the tissue like a helix. With the simplifying assumption that the two families of fibres have the same mechanical response, and that the fibre directions are symmetric with respect to the axial direction of the vessel, we have

2.2 $$k_{1}=k_{3},\;k_{2}=k_{4},\;\beta_{1}=-\beta_{2}=\beta .$$

Several conditions should be satisfied in the parameters used above in order for the model to be mathematically stable. First, *I*_4_ > 1 and *I*_6_ > 1 to show that the fibres are only engaged under tension and not compression. Furthermore, *µ* > 0, *k*_1_ > 0 and *k*_3_ > 0 to have physically meaningful interpretation, since these values translate to the slope of stress–strain curves in the principal tissue as well as fibre directions. Another assumption made in this study was tissue incompressibility, which translates to the determinant of ***F*** being unity under any deformation. This assumption is commonly made in modelling biological tissues, including arteries [12]. An additional simplification here is that the model accounts for only two families of fibres and therefore the effects of SMC, elastin and collagen are averaged within these two families.

The Cauchy stress tensor is calculated by taking the partial derivative of *W* with respect to the Lagrangian strain tensor; therefore the specific stress components are given by

2.3 $$T_{\theta }=\lambda_{\theta }\frac{\partial W}{\partial \lambda_{\theta }}\;\mathrm{and}\;T_{z}=\lambda_{z}\frac{\partial W}{\partial \lambda_{z}},$$

where *θ*,*z* denote the circumferential and longitudinal directions, respectively, and λ*_i_*, *i* = *θ,z* represent the stretch ratio in each specific direction [32].

#### 2.1.5. Data fitting

Based on the experimentally measured force and displacement values for each test, the experimental Cauchy stress and stretch ratio values were determined using the initial cross-sectional area of each specimen and assuming an incompressible tissue behaviour [12]. Subsequently, a nonlinear minimization algorithm in MATLAB (fmincon) was used to fit the constitutive parameters by fitting equation (2.3) to the experimental data while incorporating the histological analysis data. Owing to the high nonlinearity of the stress–strain relationship, converging to the ‘best-fit’ is rather difficult to achieve since it strongly depends on the initial starting point, as previously noted in [33,34]. In order to achieve the global best-fit, which should in theory be independent of the initial guess, we performed the optimization with 20 randomly selected initial starting points (MATLAB's multisearch). During this process, care was taken so that the model parameters were not constrained by the lower and upper bounds of the optimization. The sum of squared errors between the experimental data and model was used as the objective function and the corresponding *R*^2^ was used to compare the goodness of each fit.

#### 2.1.6. Statistical analysis

We performed a statistical analysis to investigate the variations in the mechanical behaviour of aortic tissue along the long axis of the vessel. In order to compare the fibre orientation of the two fibre families, we used the mean value of all samples and at all distal locations for each direction. Also in order to compare the constitutive material parameters, we studied the change in material parameters at each distal location among all samples. We used a value of *p* < 0.05 to indicate statistical significance.

## 3. Results

### 3.1. Uniaxial cyclic loading

**Figure 3.**
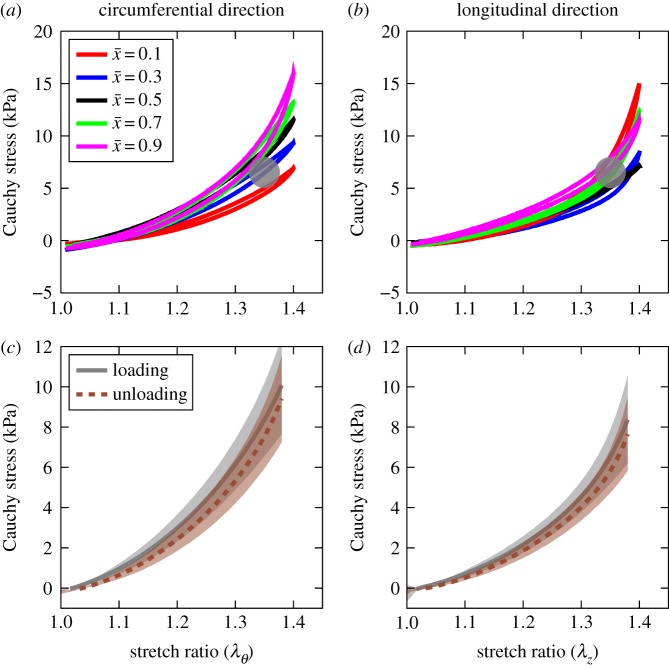
Stress–strain curves. (*a,b*) Representative hysteresis curves for circumferential and longitudinal curves for locations along the axis. The grey dots show physiological loading regime for comparison [12,35]. (*c,d*) Average and standard deviation of stress–stress curves for all the test locations for loading and unloading segments of the uniaxial tests.

Representative stress–strain curves for the final cycle of loading tests for one of the aortas are shown in figure 3 where (*a,c*) shows values for tests in the circumferential direction and (*b,d*) in the longitudinal direction. Hysteresis during the loading and unloading is evident in the stress–strain curves, indicating the well-known ‘pseudo-elasticity’ phenomenon described by Fung *et al.* [17]. According to this description, when arteries are subjected to cyclic loading, the loading and unloading branches of the stress–strain curve can be approximated by separate hyperelastic strain energy functions. The change in tissue response along the axial direction can be observed visually and is more pronounced in the circumferential direction. The stiffening behaviour of aortic tissue observed here agrees with the previously observed stiffness of the tissue in uniaxial and biaxial tissues [13,14]. We will study this effect further when we parametrize the constitutive model. The average and standard deviation of stress–strain curves for loading–unloading segments in circumferential and longitudinal directions are shown in figure 3*c*,*d*, where the anisotropic behaviour of the tissue is evident from the difference between the resulting stresses level for both loading and unloading segments. As a point of reference, the physiological loading range is also superimposed on the same graph. From this point on, we will only consider the loading segment of the stress–strain curve for modelling purposes.

### 3.2. Histology

**Figure 4.**
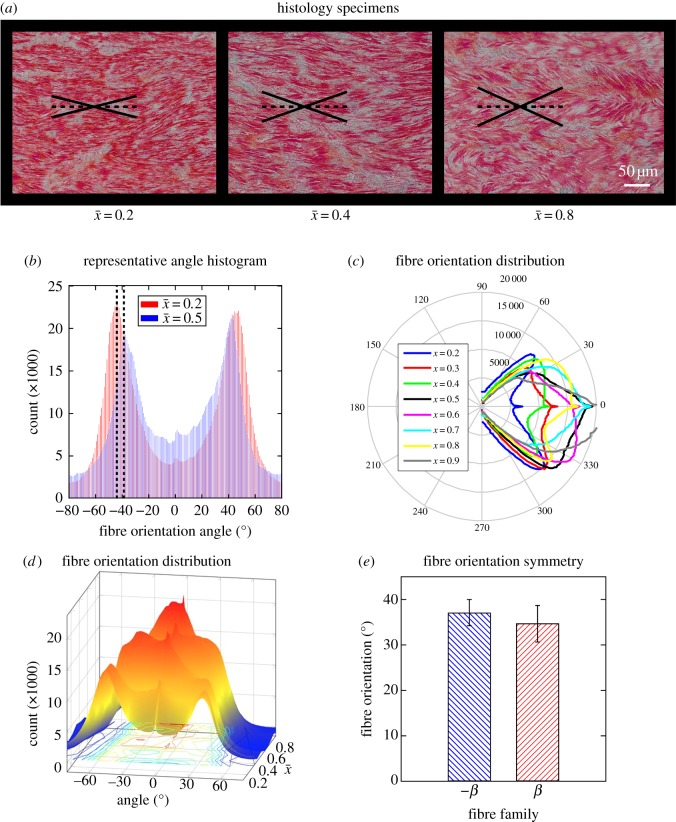
Structural analysis of aortic wall tissue from histology. (*a*) Representative histology sections of an aorta specimen at three different locations showing the fibre orientations with angles shown. (*b*) Representative histogram of angle distribution of two samples from the same aortic specimen. (*c*,*d*) Polar histogram and three-dimensional histogram of the distribution of fibre orientations as a function of axial location for one aortic specimen. (*e*) Mean bar plot of fibre orientation directions for all aortic specimens along the axis showing symmetric directions for the two fibre families.

To investigate morphological changes along the longitudinal axis, we compared histological samples extracted from various normalized positions. A representative histological sample together with the main fibre direction is shown in figure 4*a*. Collagen fibres appear as nearly straight lines in the intima and as wavy structures in the adventitia. Only the global orientations of the wavy structures were considered here for the determination of the adventitial fibre orientations [20]. The overall change in fibre orientations is visually apparent in these images, where as we move from the proximal to distal end of the aorta, the dominant direction seems to further align with the longitudinal direction. In order to determine the tissue's fibre orientation as a model parameter and also to confirm the structural assumptions made in the constitutive modelling (symmetry of the two fibre bundles, i.e. *β*_1_ = −*β*_2_, *k*_1_ = *k*_3_, *k*_2_ = *k*_4_), we performed image processing on the histological samples. This analysis confirmed our assumption and showed that fibres had a predominant direction of close to ±30–46° from the circumferential direction (figure 4*b*). Distribution of the fibre orientations is shown as a polar plot in figure 4*c*, which represents the histogram of all the samples, and as a surface plot in figure 4*d* to show spatial distribution. Furthermore, fibre orientation in the *β*_1_ and *β*_2_ directions did not show statistically significant differences, confirming the assumption of symmetry (figure 4*e*).

### 3.3. Constitutive modelling

**Figure 5.**
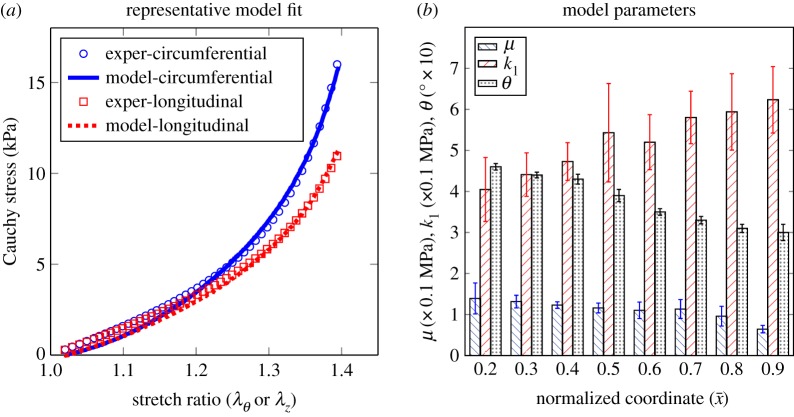
(*a*) Constitutive modelling representative fits and (*b*) mechanical properties along axial direction.

The optimized material parameters and their corresponding error are reported in table 1. The high *R*^2^-values confirm the goodness of fit for each test. As a representative, figure 5 shows the experimental and model stress–strain curves for loading and unloading in circumferential and longitudinal directions for specimen 1 at $\bar{x}=0.25.$

**Table 1.** Fitted model parameters.

$\bar{x}$	*µ* (kPa)	*k*_1_ (kPa)	*k*_2_	*θ* (°)	*R*^2^
0.2	139.2 ± 38.0	405.1 ± 78.8	2.86 ± 0.39	46 ± 0.8	0.87
0.3	132.1 ± 16.1	441.9 ± 53.6	4.55 ± 0.66	44 ± 0.7	0.89
0.4	123.1 ± 8.2	473.5 ± 46.9	4.01 ± 0.17	43 ± 1.2	0.84
0.5	116.4 ± 12.9	543.6 ± 20.1	3.11 ± 0.71	39 ± 1.5	0.85
0.6	110.6 ± 20.7	520.4 ± 67.5	4.25 ± 0.03	35 ± 0.8	0.79
0.7	113.5 ± 23.2	580.2 ± 64.5	3.26 ± 0.28	33 ± 0.9	0.88
0.8	96.4 ± 24.7	594.3 ± 93.7	2.87 ± 0.15	31 ± 1.0	0.83
0.9	64.9 ± 9.3	623.7 ± 81.1	3.65 ± 0.20	30 ± 2.0	0.91

The average material parameters reported in table 1 were then studied as a function of the normalized distance from the proximal end of the aortic samples. There was a significant decrease in the shear modulus (*µ*) as we moved from the proximal to the distal end of the blood vessel (figure 5*b*). The parameter *k*_1_, which represents the tissue stiffness along the fibre directions, showed an increase along the aortic axis, although this increase was not statistically significant (figure 5*b*). The scaling parameter *k*_2_ did not show a clear trend along the axial direction.

### 3.4. Parametric study

**Figure 6.**
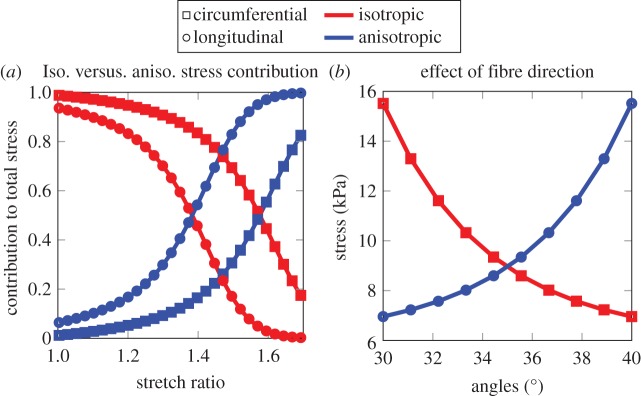
(*a*) Contribution of isotropic and anisotropic parts to total stress (equation (2.1)). (*b*) Effect of change of fibre orientation in total stress.

In addition to the analysis above, we performed a parametric study to obtain insight into the effects of the tissue anisotropy and how it contributes to the total stress induced in the tissue. First, using the material parameters given in table 1, we studied the contribution of the isotropic and the anisotropic stresses to the total stress as a function of stretch ratio. In both the longitudinal and circumferential directions, in the low strain regime (λ*_z_* < 1.4 and λ*_θ_* < 1.6), the isotropic stress seems to dominate and as the loading level increases the effect of the tissue's anisotropy becomes more pronounced (figure 6*a*). We also investigated the effect of the fibre orientations on tissue stress in the range of fibre directions determined in the previous section at λ = 1.4. As can be seen in figure 6*b*, even though this might seem a small change of angles, the results in the longitudinal and circumferential stress are significant.

## 4. Discussion

Owing to the high incidence of cardiovascular diseases and the appeal of stenting as a less invasive alternative to surgery, computational models of the aorta have become exceedingly important, especially with the ubiquity of FE models in design and animal models in the preliminary data collection stage. The mechanical response of the aorta is mainly determined by the elastin lamellae and fibre bundles that contribute to isotropic and anisotropic compliance of the tissue [36]. The main objective of this paper was to study how these mechanical properties vary in the circumferential and longitudinal directions along the aorta's axis. To achieve this goal, we performed uniaxial cyclic loading tests on aortic specimens and characterized a constitutive model for each section of tissue as a function of location.

In order to study the structure of the tissue and the effect it has on the tissue's mechanical behaviour, we used histological samples and image processing to determine local orientation of fibre bundles. Depending on the location of the specimen, histological images showed significant changes in the tissue's fibre orientation, while maintaining a predominance of two fibre families. As we move from the proximal end of the aorta to the distal end, fibre bundles tend to align themselves more with the circumferential direction of the tissue and therefore show less compliant behaviour in circumferential stretching. Despite this effect, the fibre bundles were principally symmetric across the longitudinal axis of the aorta with a helical shape. The symmetry of the fibre bundles supports using an orthotropic material model.

To model the tissue behaviour under various loading conditions, we used stress–strain curves from uniaxial tests and simultaneously fitted a nonlinear model to the experimental results from both circumferential and longitudinal protocols. Results showed stiffer mechanical behaviour in the circumferential direction, which was reported previously in the literature [14,25,31]. In addition, tissue exhibited stiffer behaviour in the circumferential direction as we tested specimens from more distal locations. The same tests in the longitudinal direction did not show a significant trend for different locations. In a separate modelling step, we implemented parameter fitting to the stress–strain data to directly fit the fibre directions without including the histological data of the fibre orientations. The results showed very similar fibre directions (30–50°) to those determined through histological examination, which could be taken as an indication for the robustness of inclusion of fibre orientations in the constitutive model.

Despite good agreement between our model and experimental data, there are several limitations associated with our study. First, while we repeated uniaxial tests in the longitudinal and circumferential directions and used both results in the modelling step, the mechanical loading on the tissue in physiological states is mostly biaxial [37,38]. Including biaxial stress–strain information in addition to fibre orientations could increase the accuracy and applicability of the model. Furthermore, the tissue's mechanical behaviour is determined not only by the orientation of fibre bundles but also by the density of such structural elements. Higher elastin content proximally and collagen content distally could describe the stiffer behaviour of the tissue. On a separate set of parameter fits, we included the dispersion variable (*ρ* in [31]). Our analysis showed this parameter to be very close to 1 for most cases, which could indicate a near homogeneous distribution of the fibres. To simplify calculations, we excluded this parameter from our analysis. Finally, aorta consists of three distinct layers, each with different material characteristics that engage in different loading conditions. In our study, we used a single layer formulation and therefore averaged the effect of the three layers. Although this simplification is commonly seen in modelling aortic tissue [19], it should be noted that it reduces the structural relevance of our model to a more phenomenological model.

In summary, this study quantified the local variations of the microstructural properties of the aortic tissue and how they affect its mechanical properties. An advantage of including structural information into the constitutive model is that it allows for the observation that collagen fibres show less influence on the mechanical response of the tissue at low luminal pressures [11,20]. In addition, this model not only takes into account the stress–strain behaviour of the whole tissue and therefore gives average isotropic properties of the tissue, but it also accounts for the structural character of the tissue since it takes into account underlying histology of the aortic tissue as detailed in the previous section.

## Data accessibility

Our data will be available at http://purl.stanford.edu/gv632sp8194.
